# UT-B-deficient mice develop renal dysfunction and structural damage

**DOI:** 10.1186/1471-2369-13-6

**Published:** 2012-01-30

**Authors:** Lei Zhou, Yan Meng, Tianluo Lei, Dan Zhao, Jing Su, Xuejian Zhao, Baoxue Yang

**Affiliations:** 1 Prostate Diseases Prevention and Treatment Research Center, Department of Pathophysiology, Norman Bethune College of Medicine, Jilin University, Changchun, China; 2 Department of Pharmacology, School of Basic Medical Sciences, Peking University, Beijing, China; 3 Department of Pathology, Affiliated Hospital to Changchun University of Chinese Medicine, Changchun, China

**Keywords:** urea transporter, renal function, kidney, UT-B, knock-out

## Abstract

**Background:**

Urea transporter UT-B is the major urea transporter in erythrocytes and the descending vasa recta in the kidney. In this study, we investigated the effects of long-term UT-B deficiency on functional and structural defect in the kidney of 16-and 52-week-old UT-B-null mice.

**Methods:**

UT-B-knockout mice were generated by targeted gene disruption and lacked UT-B protein expression in all organs. The urinary concentrating ability of mice was studied in terms of daily urine output, urine osmolality, and urine and plasma chemistries. Changes in renal morphology were evaluated by hematoxylin and eosin staining.

**Results:**

The UT-B-null mice showed defective urine concentrating ability. The daily urine output in UT-B-null mice (2.5 ± 0.1 ml) was 60% higher and urine osmolality (985 ± 151 mosm) was significantly lower than that in wild-type mice (1463 ± 227 mosm). The 52-week-old UT-B-null mice exhibited polyuria after water deprivation, although urine osmolality was increased. At 52 weeks of age, over 31% of UT-B-null mice exhibited renal medullary atrophy because of severe polyuria and hydronephrosis.

**Conclusions:**

Long-term UT-B deficiency causes severe renal dysfunction and structural damage. These results demonstrate the important role of UT-B in countercurrent exchange and urine concentration.

## Background

In mammals, most nitrogenous waste is excreted in urine in the form of urea. Urea represents about 40-50% of all urinary solutes in humans, and even more in rodents [1,2]. Compared with the blood levels of sodium and chloride, the blood urea level is relatively low (5-10 mmol/l with a normal-protein diet). The urinary urea concentration may be 20-100 times higher than that in the blood in humans, and up to 250 times higher in mice. NaCl is usually not, or only modestly, concentrated in the urine (up to twice the plasma level). Potassium, which is actively secreted in the collecting duct lumen, is usually concentrated by 5-30 times the plasma level. Thus, the solute concentrating effort of the kidney is mainly devoted to concentrating urea [1,2]. Urea concentration is highly dependent on intrarenal urea recycling and selective urea permeability, which are mediated by urea transporters (UT) in the kidney [3,4].

UT-B is highly expressed in erythrocytes and in the endothelium of the descending vasa recta throughout the renal medulla [5]. In humans, UT-B has been shown to carry the Kidd blood group antigen [6-9]. Mutations in human UT-B significantly decrease urea permeability in erythrocytes [9-11] and result in mild urinary concentrating defects [12]. UT-B-null mice, which also have a urea-selective urine concentrating defect, offer an animal model to characterize the role of UT-B in renal function, particularly urinary concentration [13].

The contribution of UT-B to countercurrent exchange has been confirmed by comparing the responses to an acute urea load and a chronic high-protein diet between UT-B-null mice and wild-type mice [11]. That study showed that the plasma urea level was significantly higher whereas the urine urea level was significantly lower in UT-B-null mice than in wild-type mice fed a normal protein diet. Acute urea infusion or chronic high protein intake in UT-B-null mice did not increase the urinary urea concentration and failed to increase the concentration of non-urea solutes, as observed in wild-type mice. These previous results suggest that intrarenal urea recycling is very dependent on urea countercurrent exchange, mostly involving UT-B [11,14]. However, the long-term effects of impaired urea countercurrent exchange on renal function and morphology are currently unclear.

Therefore, the goal of this study was to determine the effects of long-term UT-B deficiency on renal function and morphology in mice. In brief, we found that 52-week-old UT-B-null mice exhibited remarkable polyuria, although their urine concentrating ability was increased by water deprivation. About 31% of the 52-week-old UT-B-null mice had severe hydronephrosis and renal morphological abnormalities. Taken together, the results of this study indicate that long-term polyuria caused by UT-B deficiency can result in severe renal functional and structural damage. Our data provide evidence that UT-B plays an important role in the urine concentrating mechanism.

## Methods

### Mice

Transgenic UT-B-null mice were generated by targeted gene disruption as previously reported [13]. UT-B-null mice did not express detectable UT-B protein in any organ. Female wild-type and UT-B-null mice at 16 and 52 weeks of age were used in this study. All mice had a C57BL genetic background. Measurements were done in litter-matched mice produced by intercrossing heterozygous mice. All animal procedures were approved by the Jilin University Committee on Animal Research.

### Studies of urinary concentrating ability

Daily urine output was evaluated in mouse metabolic cages (Harvard Apparatus, Holliston, MA). In some experiments, urine samples were obtained from the same mice under basal conditions (with unrestricted access to food and water) and after 18 h of food and water deprivation. Urine osmolality was measured by freezing point osmometry (micro-osmometer, Precision Systems Inc. Natick, MA). Blood samples were obtained from the tail vein. Urine and plasma chemistries were measured by the Clinical Chemistry Laboratory of Jilin University, China.

### Acute urea load

Adult female wild-type and UT-B-null mice (5 mice/group, body weight 22-25 g) were adapted to metabolic cages for 3 days before the urea-load experiment. The acute urea-load experiments were done as previously described [11]. In brief, during the acute urea-load experiment, urine was collected every 2 h after spontaneous voiding and/or bladder massage. The lower part of the metabolic cage was removed and replaced by a tray covered with Parafilm. Mouse bladders were emptied by gentle abdominal massage. To avoid evaporation, the Parafilm sheets were inspected every 10 min, and any urine found on the sheets was collected and placed in preweighed tubes containing a small amount of paraffin oil to prevent evaporation. Urine collected from 8:00 to 10:00 am was defined as the basal period. The urea load was administered just after urine collection at 10:00 am. Each mouse received an intraperitoneal injection of 300 μl of 1 M urea solution, corresponding to 300 μmol of urea, about 10% of the daily urea excretion. Urinary volume, urea and osmolalilty were measured. The urinary urea concentration was measured with a urea assay kit (BioAssay Systems. Hayward, CA).

### Renal Morphology

Kidneys were fixed *in situ *by perfusion with 4% paraformaldehyde in phosphate-buffered saline. Fixed tissues were processed by routine histological methods, and 6-μm-thick paraffin sections were stained with hematoxylin and eosin.

### Statistical analysis

Results obtained in UT-B-null mice were compared with those in wild-type mice by Student's *t *test. One-way analysis of variance (ANOVA) was used to compare three groups, followed by Fischer's *post hoc *test. One-way repeated-measures ANOVA was used to compare both genotypes in the dehydration study. Values of P < 0.05 were considered statistically significant.

## Results

**Figure 1:**
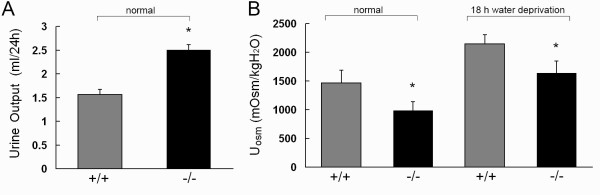
**Urine concentrating ability in 52-week-old UT-B-null and wild-type mice**. *A*: Twenty-four hour urine output (mean ± SE, 5 mice/group). *B*: Urine osmolality measured in mice given free access to food and water (*basal*) and after water deprivation for 18 h. *p < 0.01 versus wild-type mice.

The urine concentrating ability of 52-week-old mice was studied in metabolic cages. As previously reported [13], UT-B deficiency resulted in a significant urinary concentrating defect. Figure 1A shows the daily urinary output. At the age of 52 weeks, UT-B-null mice excreted about 60% more fluid than did wild-type mice of the same age. The urine osmolality in UT-B-null mice (985 ± 151 mosm) was significantly lower than that in wild-type mice (1463 ± 227 mosm, Figure 1B). After water deprivation for 18 h, the urine osmolality was increased in UT-B-null mice (1636 ± 213 mosm) and wild-type (2143 ± 165 mosm), which indicates that the urine concentrating ability is defective in UT-B-null mice. The plasma urea concentration was 52% higher in UT-B-null mice compared with wild-type mice (Table 1), and was higher in 52-week-old UT-B-null and wild-type mice compared with 16-week-old mice. The urine/plasma (U/P) concentration ratio for creatinine was about 26% lower in UT-B-null mice than in wild-type mice at the age of 52 weeks, whereas the U/P concentration ratio for urea was 59% lower in UT-B-null mice than in wild-type mice (Table 1). These results were consistent with those reported for younger UT-B-null mice [15]. Plasma creatinine and creatinine clearance (an index of the glomerular filtration rate) were similar in wild-type and UT-B-null mice, suggesting that aging did not impact on glomerular hemodynamics. Urea clearance was 34% lower in UT-B-null mice than in wild-type mice (Table 1). In contrast, the clearance of sodium, potassium and chloride was similar in both groups of mice (data not shown).

**Table 1:** Water and solute handling.

		16 weeks old			52 weeks old	
	
	Wild-type	UT-B-null	Relative difference (KO/WT)	Wild-type	UT-B-null	Relative difference (KO/WT)
**Body weight, g**	24.12 ± 1.82	24.42 ± 1.10	1.01	37.08 ± 1.77	36.26 ± 2.31	0.98
**Hydronephrotic kidneys/total kidneys**	0/45	0/42		0/33	12/39	
**Urine output, ml/day**	2.41 ± 0.08	4.05 ± 0.13*	1.68	1.56 ± 0.11	2.5 ± 0.11^#^	1.6
**Urinary osmolality**, **mosm/kg H_2_O**	1622 ± 87	1012 ± 239*	0.62	1463 ± 227	985 ± 151^#^	0.67
**Osmolar excretion, μmol/L**	3741 ± 314	4171 ± 792	1.11	2295 ± 400	2533 ± 281	1.1
**Plasma creatinine, μmol/L**	29.6 ± 2.54	32 ± 2.02	1.08	28.53 ± 5.20	29.06 ± 1.29	1.02
**Urinary creatinine, mmol/L**	2.49 ± 0.42	1.20 ± 0.10*	0.48	3.28 ± 0.18	2.62 ± 0.25^#^	0.8
**U/P creatinine**	86.5 ± 17.6	37.9 ± 5.0*	0.44	121.0 ± 16.2	90.1 ± 5.6	0.74
**Creatinine clearance, ml/day**	198.1 ± 39.3	156.8 ± 22.1	0.79	188.1 ± 32.3	233.7 ± 23.9	1.24
**Plasma urea, mmol/l**	10.94 ± 0.88	13.02 ± 0.63*	1.19	15.36 ± 3.32	23.39 ± 6.22^#^	1.52
**Urinary urea, mmol/L**	904 ± 20	545 ± 15*	0.6	755 ± 24	475 ± 10^#^	0.63
**Urea excretion, μmol/day**	2083 ± 125	2249 ± 127	1.08	1166 ± 55	1228 ± 63	1.05
**Urea clearance, ml/day**	198.3 ± 26.3	169.5 ± 10.2*	0.85	76.7 ± 8.3	50.4 ± 5.8^#^	0.66
**U/P Urea**	82.63 ± 7.61	41.86 ± 2.12*	0.51	49.15 ± 1.85	20.31 ± 1.36^#^	0.41

Values are means ± SE of 5 mice/group. U/P: urine/plasma. Comparisons were made using Student's *t*-test. *P < 0.05 versus 16-week-old wild-type mice, ^#^*P *< 0.05 versus 52-week-old wild-type mice.

**Figure 2:**
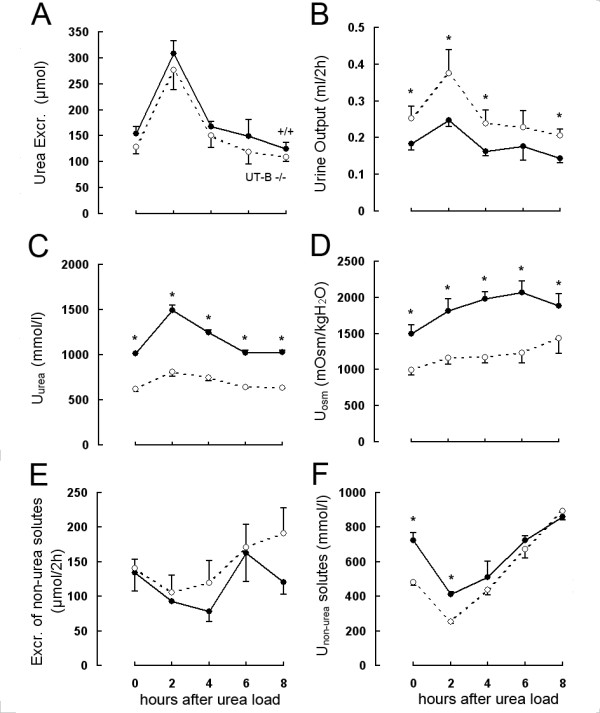
**Effect of acute urea loading on the urinary concentrating ability and renal urea handling in 52-week-old UT-B-null and wild-type mice**. An acute urea load (300 μmol) was injected intraperitoneally after the first 2-h urine collection (*time 0*). *A*: Urea excretion. *B*: Urinary output. *C*: Urinary urea concentration (U_urea_). *D*: Urinary osmolality (U_osm_). *E*: Excretion of non-urea solutes. *F*: Non-urea solute concentration (U_non-urea _solutes). Two-way ANOVA (genotype and 2-h periods) indicated significant differences between the two genotypes at the indicated times (Fisher's post hoc test).

To evaluate the contribution of UT-B to the excretion of a urea load, UT-B-null and wild-type mice were given an acute urea load of 300 μmol. Urea excretion increased within the first 2 h after urea administration in both groups in a very similar manner (Figure 2A). However, these increases were associated with markedly different changes in urinary flow rate (Figure 2B) and urea concentration (Figure 2C) between the two genotypes. Wild-type mice showed increases in urea concentration and urinary osmolality after the first post-load period and with a very small increase in urinary flow rate. In contrast, the urinary flow rate in UT-B-null mice almost doubled with only modest increases in urinary urea concentration and urinary osmolality (Figure 2C, D). The cumulative amount of urine excreted above the basal level during the first 4 h after the urea load was 614 ± 98 μl in UT-B-null mice compared with 408 ± 24 μl in wild-type mice at the age of 52 weeks. The excretion of non-urea solutes remained almost unchanged in the two groups (Figure 2E). Administration of exogenous urea also improved the ability of the kidney to concentrate other urinary solutes (non-urea solutes) in both wild-type and UT-B-null mice (Figure 2F).

**Figure 3:**
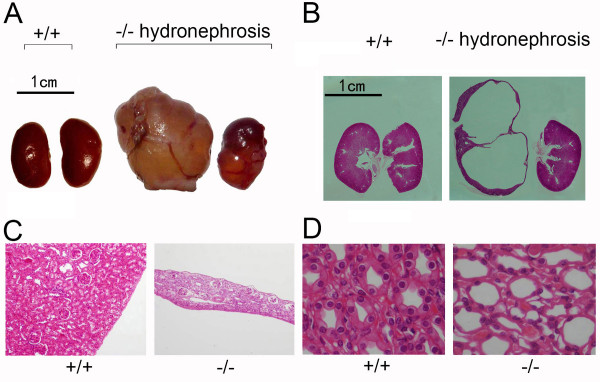
**Renal morphology of UT-B-null mice**. *A: *Gross structure of kidneys from wild-type (left) and UT-B-null (right) mice. *B: *Paraffin-embedded sections of a normal kidney (*left*) and a hydronephrotic kidney (*right*). *C*: Medullary damage and cortex thinning *(right) *are apparent on micro-morphologic images of the UT-B-null kidney but not in the wild-type kidney *(left)*. *D: *High magnification images show collecting duct dilation in the UT-B-null kidney (*right*) but not in the wild-type kidney (*left*).

Some 52-week-old UT-B-null mice (~30%), but no wild-type mice of the same age, showed marked tumor-like swelling of the bilateral flanks. Flank swelling was found to be caused by kidney enlargement (Figure 3A) as the weight of the kidney in UT-B-null mice (7.54 ± 0.27 kidney/body weight) were significantly greater than those in wild-type mice (6.8 ± 0.27) at the age of 52 weeks (P < 0.05).

Morphological evaluation of the kidneys from wild-type mice showed a well demarcated cortex and papilla. In contrast, the kidneys from UT-B-null mice (Figure 3B, C) showed medullary atrophy and cortical thinning. Some 52-week-old mice had hydronephrosis and markedly enlarged and transparent kidneys. Tissue morphological assessment also showed dilated renal collecting ducts in 52-week-old UT-B-null mice (Figure 3D), but not in wild-type mice at the same age.

## Discussion

UT-B deletion impairs urea concentration in the medullary interstitium and leads to defective countercurrent exchange. These outcomes decrease the osmolality of fluid in the medullary interstitium. The urinary concentrating defect in UT-B-null mice demonstrates the importance of the vasa recta in countercurrent exchange. The vasa recta seems to more important in intrarenal urea recycling than the thin descending limb of Henle based on data obtained from UT-B- [11,13,14] and UT-A2- [16] null mice. Older UT-B-null mice experience long-term urea-selective urine concentration defects and elevated blood urea concentrations, which may lead to functional and morphological abnormalities. Indeed, we have already reported heart block in 52 weeks old UT-B-null mice [17]. Here, we add to those findings by reporting marked functional and morphological defects in the kidney in 52-week-old UT-B-null mice.

The present study showed that the urinary concentrating ability in 52-week-old UT-B-null mice was similar to that in 16-week-old UT-B-null mice, although urine output was reduced in both genotypes at 52 weeks of age compared with that at 16 weeks of age. Interestingly, urine osmolality 52-week-old UT-B-null mice increased substantially following water deprivation, albeit to a level just lower than that in water-deprived 16-week-old UT-B-null mice. Taken together, these results indicate that older UT-B-null mice experience long-term urine concentrating defects. However, fluid turnover and the concentrations of the major plasma solutes were unchanged in older versus younger UT-B-null mice.

Similar to a previous report [11], the administration of a urea load increased urea excretion to a similar extent in UT-B-null and wild-type mice, although this was associated with markedly different changes in urine flow rate and urinary urea concentration between the two genotypes. The urinary urea concentration and urinary osmolality increased in wild-type mice after the urea load, with a slight increase in urine flow rate, which was probably due to greater sequestration of urea in the inner medulla. Urine output decreased in wild-type mice to less than 50% of the basal level. In contrast, the urinary urea concentration and urine osmolality increased modestly, while the urine flow rate was significantly increased compared with the basal level in 52-week-old UT-B-null mice, because of the urine concentrating defect.

Almost one-third of the 52-week-old UT-B-null mice exhibited marked tumor-like swelling of the bilateral flanks. This flank swelling was caused by kidney enlargement as morphological examination of the kidneys from UT-B-null mice showed medullary atrophy and cortical thinning. Similar changes in renal morphology have been seen in aquaporin-null mice, which exhibit polyuria as a result of increased intrarenal pressure [15,18]. Most of the 52-week-old UT-B-null mice had dilated collecting ducts in the renal cortex and medulla, which may result from long-term polyuria, similar to that in aquaporin 2-null mice [18]. The age-dependent medullary atrophy in UT-B-null mice suggests that long-term urine concentrating defects cause marked renal structural damage over time.

## Conclusions

In conclusion, our results indicate that long-term UT-B deficiency causes severe renal dysfunction and structural damage, revealing important roles of UT-B in countercurrent exchange and urine concentration.

## Competing interests

The authors declare that they have no competing interests.

## Authors' contributions

LZ, YM, TL, DZ, JS performed the experiments, LZ, TL, YM, XZ, BY analyzed the data, LZ, TL, BY interpreted the results of the experiments, LZ, BY prepared the figures for publication, LZ, YM, BY drafted the manuscript, LZ, YM, TL, BY revised the manuscript, LZ, YM, TL, DZ, JS, XZ, BY approved the final version of the manuscript, XZ, BY conceived and designed the study.

## Pre-publication history

The pre-publication history for this paper can be accessed here:

http://www.biomedcentral.com/1471-2369/13/6/prepub
